# Synthesis and characterization of polyphosphazene microspheres incorporating demineralized bone matrix scaffolds controlled release of growth factor for chondrogenesis applications

**DOI:** 10.18632/oncotarget.23304

**Published:** 2017-12-14

**Authors:** Bo Ren, Xiaoqing Hu, Jin Cheng, Zhaohui Huang, Pengfei Wei, Weili Shi, Peng Yang, Jiying Zhang, Xiaoning Duan, Qing Cai, Yingfang Ao

**Affiliations:** ^1^ Institute of Sports Medicine, Peking University Third Hospital, Beijing Key Laboratory of Sports Injuries, Beijing 100191, China; ^2^ State Key Laboratory of Organic-Inorganic Composites, Beijing Laboratory of Biomedical Materials, Beijing University of Chemical Technology, Beijing 100029, China

**Keywords:** polyphosphazenes microsphere, demineralized bone matrix, control release, growth factor, chondrogenesis

## Abstract

As a promising strategy for the successful regeneration of articular cartilage, tissue engineering has received increasing recognition of control release. Two kinds of functional poly (alanine ethyl ester-co-glycine ethyl ester) phosphazene microspheres with different ratios of side-substituent groups were synthesized by emulsion technique. The rate of degradation/hydrolysis of the polymers was carefully tuned to suit the desired application for control release. For controlled delivery of growth factors, the microspheres overcame most of severe side effects linked to demineralized bone matrix (DBM) scaffolds, which had been previously optimized for cartilage regeneration. The application of scaffolds in chondrogenic differentiation was investigated by subcutaneous implantation in nude mice. In the present study, we have provided a novel microsphere-incorporating demineralized bone matrix (MS/DBM) scaffolds to release transforming growth factor-β1 or insulin-like growth factors-1. Laser confocal fluorescence staining showed that the surface of microspheres was a suitable environment for cell attachment. Histological and immunohistochemical evaluations have shown that significantly more cartilaginous extracellular matrix was detected in MS/DBM group when compared with DBM alone group (P<0.05). In addition, the biomechanical test showed that this composite scaffold exhibited favorable mechanical strength as a delivery platform. In conclusion, we demonstrated that MS/DBM scaffolds was sufficient to support stem bone marrow-derived mesenchymal stem cells chondrogenesis and neo-cartilage formation.

## INTRODUCTION

Articular cartilage has a relatively high incidence of damage from sports injury and poor self-repair capacity [1]. During the development of regenerative medicine approaches for cartilage tissue repair, the main focus has been the implantation of cell-containing biomaterial into the defect area, with the aim to promote the differentiation of stem cells into chondrocytes. Previous studies have indicated that cartilage tissue repair needs two basic requirements including 3D structure supporting chondrogenesis and favorable biocompatibility for cells metabolism activity [2]. Since the main component in cartilage-specific extracellular matrix (ECM) is collagen, we prepared a demineralized bone matrix (DBM) scaffold which is mainly consisted by collagen via bone tissue demineralization [3]. It is an attractive biomaterial in tissue engineering because it has excellent biodegradability and does not elicit adverse immune responses [4]. Our studies recently demonstrated successful BMSC infiltration and survival in DBM scaffold when using DBM to repair cartilage defects *in vivo* [5].

Besides the scaffold, the integration and stability of the engineered cartilage were also influenced by cytokines released from implantation microenvironment, of which growth factors (GFs) is able to control cellular fate [6]. Various strategies are available when considering how to modify the local concentration of GFs within the articular cartilage defect [7]. At present, development of multifunctional polymeric carriers for GFs delivery is believed to be a promising approach to promote tissue homeostasis and regeneration. Among these studies, numerous researchers have demonstrated that additional GFs release can enhance cartilage regeneration, such as transforming growth factor-beta 1 (TGF-β1) and insulin-like growth factors-1 (IGF-1), which are key mediators in promoting chondrogenesis [8–10]. However, the short release period and low biocompatibility are still major obstacles for clinical application [11]. One option to enhance the efficacy of GF is to incorporate them into polymeric biomaterials maintain their bioactive stability and control release. Safety of microsphere approach has been evaluated in clinic and certain efficacy has been shown [12, 13]. Therefore, strategies based on microspheres combined with scaffolds offer further hopes for consistently and stably promoting chondrogenesis.

Microspheres is a well-known medium to control release bioactive factors. However, efforts are still needed to overcome problems after cell transplantation such as low survival rate, poor differentiation and integration ability into matrix deposition [14]. Over the past decades, researchers commonly focused on poly(lactide-co-glycolide) and polycaprolactone as material to prepared microspheres [12, 15]. Considering the biological activity in supporting tissue development, one of the most important issues involved in regenerative engineering is how stem cells react when they are in contact with materials. In concerning about the stimulation and adverse effects of acidic degradation products of polyester, another potential biomaterial appeared to be a simple target [16]. Poly (alanine ethyl ester-co-glycine ethyl ester) phosphazene (PAGP) is biodegradable polymer whose backbone consist of alternating phosphorus and nitrogen atoms [17]. It provides a unique platform for developing advanced materials for biological applications as they combine an intrinsic biodegradability with a versatile synthetic route, which allows for structural diversity [18]. Because its organic substituents are linked to the phosphorus atoms as side groups, its degradation products under physiological conditions contain compounds including phosphate, ammonium and amino acids [19]. Moreover, its nontoxic hydrolysis products have unique buffering ability and low toxicity to human body. Therefore, this material appears to be an ideal candidate for biomedical applications.

In this study, we chose biodegradable PAGP material to synthesize two kinds of functional microspheres, and investigated their release pattern depends on predictable degradation rate. After that, we evaluated a novel formulation of PAGP microspheres releasing TGF-β1 or IGF-1 and incorporated DBM scaffolds followed by subcutaneous implanting in nude mice to support chondrogenic differentiation of BMSCs and induce cartilage matrix formation.

## RESULTS

### Synthesis and characterization of PAGP polymers

**Figure 1:**
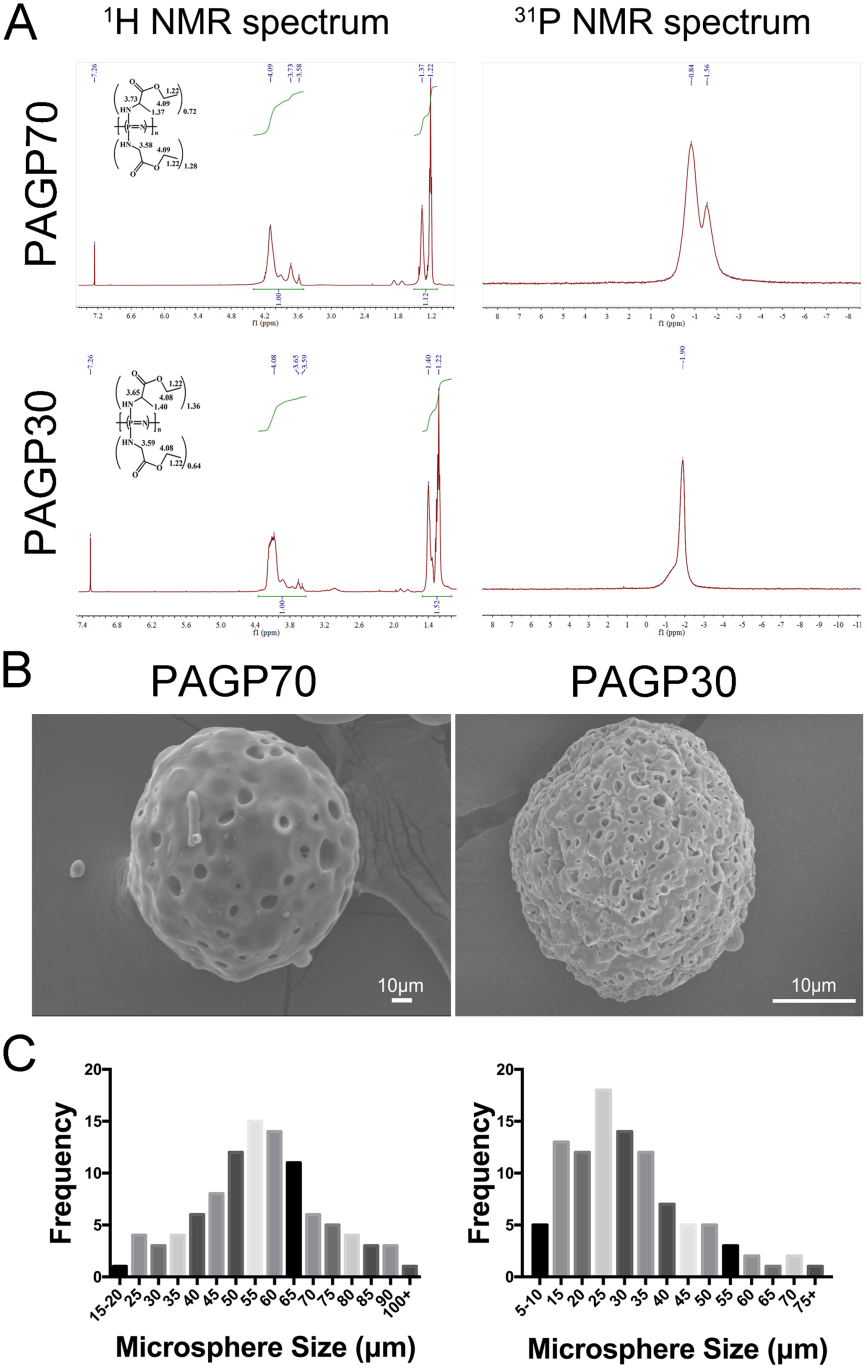
Characterization of PAGP microspheres **(A)** ^1^H and ^31^P NMR spectrum of PAGP70. **(B)** Morphological images of PAGP microspheres degradation obtained by scanning electron microscope (SEM) (500X, scar bar = 50 μm or 20 μm respectively). **(C)** The diameter distribution of microspheres was analyzed by Image J software (PAGP70, left; PAGP30, right).

Chemical structures of the obtained PAGP polymers were characterized by ^31^P and ^1^H nuclear magnetic resonance (NMR) spectra. As presented in Figure 1A, all the characteristic signals related to phosphorus atom in backbone and hydrogen protons in relating side groups were identified. Notably, the appearance of chemical shifts at 1.22, 1.40, 3.59, 3.65 and 4.08 on ^1^H spectrum confirmed the successful incorporation of alanine ethyl ester and glycine ethyl ester onto the polymer backbone (Table 1). The grafting ratio of alanine ethyl ester was calculated to be 68% for PAGP70 and 36% for PAGP30. The two polymers were synthesized from the same batch of PDCP, however, PAGP30 displayed lower intrinsic viscosity than PAGP70. The reason is that the viscosity is closely related to polymeric chain movement, and alanine ethyl ester brings higher hindrance to polyphosphazene backbone than glycine ethyl ester. The two side groups are also different in hydrophilicity, therefore, properties of the resulting PAGP materials are highly dependent on their ratios.

**Table 1:** Identification of the properties of synthesized PAGP

Sample	^31^P(ppm)^a^	^1^H(ppm)^a^	Intrinsic viscosity (dL/g)	Yield (%)	Mol% Ala^b^	Mol% Gly^b^
PAGP70	-1.90	1.22 (6H, Gly, Ala), 1.40 (3H, Ala), 3.59 (2H, Gly), 3.65 (1H, Ala), 4.08 (4H, Gly, Ala)	87.4	51.2	68	32
PAGP30	-0.84	similar as PAGP70	51.3	48.3	36	64

^a^ NMR spectra were obtained by dissolving PAGP polymers in CDCl_3_.

^b^ Ala refers to alanine ethyl ester, Gly refers to glycine ethyl ester.

### Morphological characterization of PAGP microspheres

The morphological difference between two PAGP microspheres was identified according to SEM images. The PAGP70 microspheres were generally spherical, and many irregular pores were formed on the smooth external surface (Figure 1B). In comparison, PAGP30 microspheres exhibited a much rougher surface with more intensive pores. On the other hand, microspheres showed a distribution size ranging from 10 to 100 μm, as presented in the histogram plot (Figure 1C). The average diameter was 54.22±19.19 μm for PAGP70 and 34.11±18.82 μm for PAGP30.

### PAGP microsphere degradation and control release pattern

**Figure 2:**
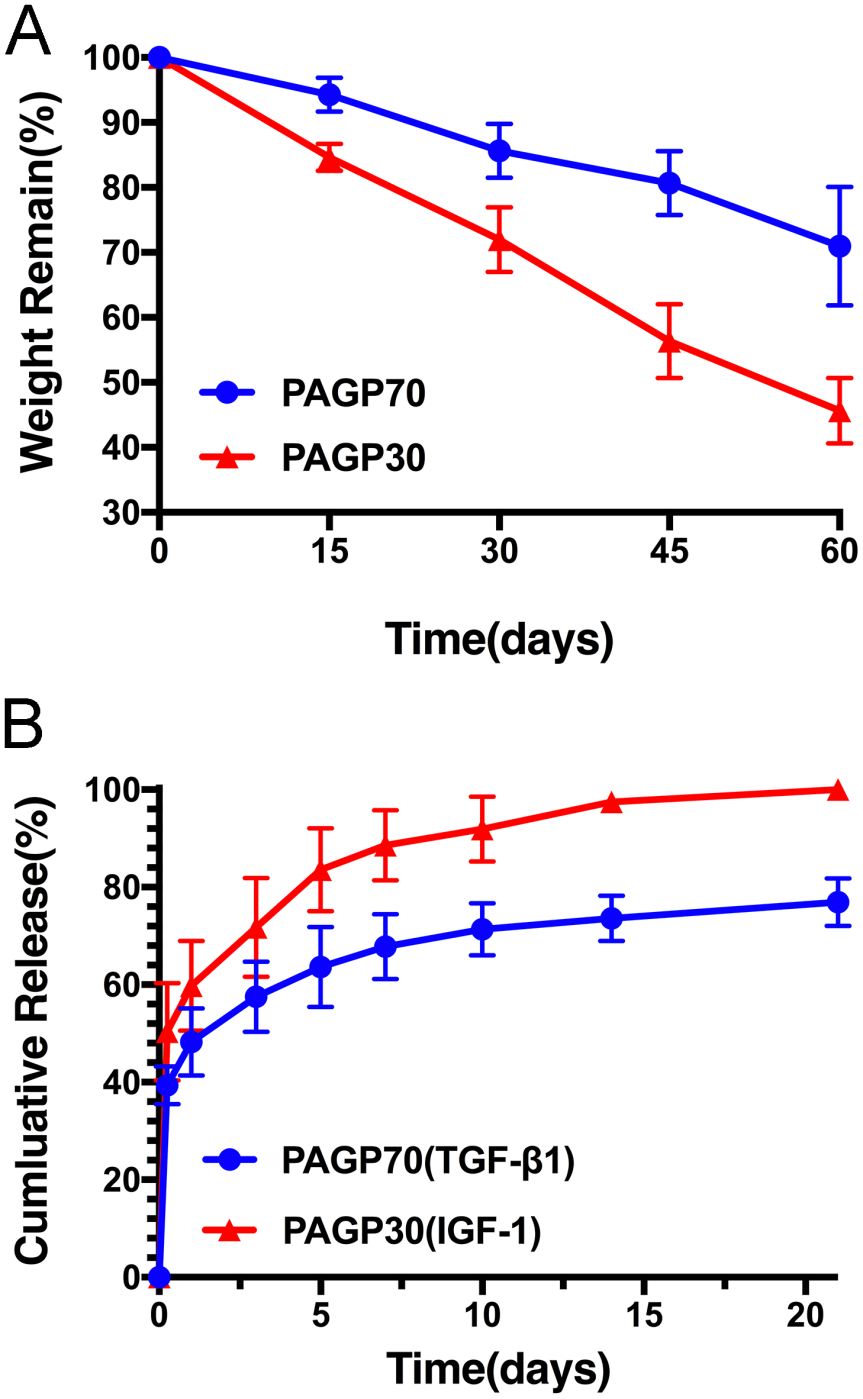
Microspheres degradation and release characteristics **(A)** Weight loss of microspheres in PBS solutions over 60 days. **(B)** The percentage of cumulative release of TGF-β1 from PAGP70 microsphere and IGF-I from PAGP30 microsphere was determined by ELISA over 21 days.

Hydrolysis of the PAGP microspheres was conducted in phosphate buffer saline (pH 7.4) at 37°C as long as 60 days. It showed that both PAGP microspheres displayed a gradual weight loss along with a longer soaking time (Figure 2A). After 60 days of hydrolysis, the weight loss of PAGP70 and PAGP30 reached ∼ 30 wt.% and ∼ 55 wt.%, respectively. These results demonstrated that the change of polymer weight occurs following the decreasing in polymer molecular weight degradation. The PAGP polymer would be liable to hydrolyze if it had higher content of glycine ethyl ester, which is more hydrophilic than alanine ethyl ester.

Previous studies had revealed the final optimal concentration of TGF-β1 (10 ng/mL) and IGF-1 (100 ng/mL) [20, 21]. Therefore, the loading dosage of IGF-1 utilized in this study was significantly higher than TGF-β1. Meanwhile, after cartilage damage, the expression of TGF-β1 significantly increased in cartilage layers and maintained at a relatively high level approximately until day 14, the level of IGF-1 increased during the first 3 days and showed a gradual decrease afterward [22, 23]. Due to these reasons, we chose the PAGP30 microsphere which smaller and degraded faster as the carrier for IGF-1 so that it had an obvious burst release, while PAGP70 as the carrier for TGF-β1, which promised a slower release of it. Finally, according to the calculation of loading capacity per unit microsphere, 90.34 ng/mg of TGF-β1 was incorporated into PAGP70 microspheres and 569.57 ng/mg of IGF-1 was incorporated into PAGP30 microspheres respectively. The GFs release kinetics curves detected by ELISA assay were shown in Figure 2B, and it was related to microsphere degradation. The release of TGF-β1 encapsulated in PAGP70 microspheres was mainly consisted of a burst release (48.24±6.91%) within 1 day, and followed by a slower sustained release (76.9±4.93%) until 21 days. On the other hand, the IGF-1 encapsulated in PAGP30 microspheres displayed relatively rapid release behavior on the first day (59.75±9.22%) and a higher cumulative release (97.52±1.16%) within 14 days. In addition, the ELISA methods used in this study could only detected proteins by forming immune complexes in antibody/ antigen recognition reactions. This method may partially account for the maintenance of the bioactivity of growth factors for up to 21 days.

### BMSCs culture and PAGP biocompatibility

**Figure 3:**
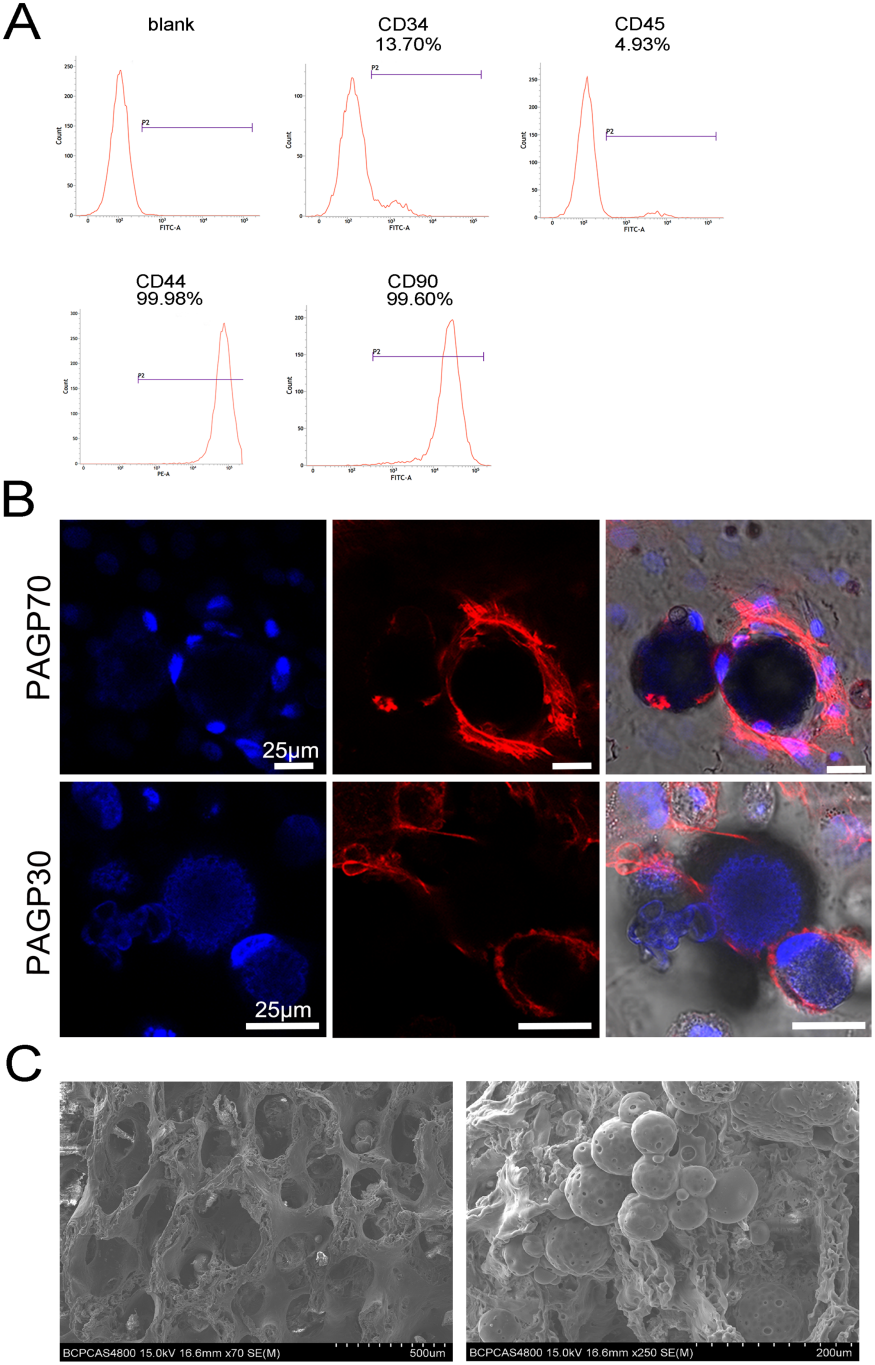
The PAGP biocompatibility and preparation of composite MS/DBM scaffolds **(A)** The results of flow cytometry showed that BMSCs had specific surface antibody markers. **(B)** Laser confocal microscopic images of the existence of variable BMSCs on the surface of PAGP microsphere after monolayer cultured 24h *in vitro* (Red, Rhodamine phalloidin stained cytoskeleton; Blue, Hoechst 33258 stained nuclei; scale bar= 25 μm). **(C)** Morphological images of MS/DBM scaffolds by scanning electron microscope (SEM) (500X, scar bar = 500 μm and 200 μm respectively).

Flow cytometry results indicated that BMSCs at passage two (P2) exhibited positive phenotypic markers CD44 (99.98%) and CD90 (99.60%), while the expression of lipopolysaccharide receptor CD34 and leukocyte common antigen CD45 was negative (13.70% and 4.93%, respectively) (Figure 3A). It was also shown that BMSCs had homogeneous phenotype after isolation and expansion. The initial cellular response to the material surface is a necessary part of early preclinical screening. Using unloading microspheres, we investigated the impact of PAGP material on BMSCs proliferation. Laser confocal microscopic images revealed that BMSCs grew well on the surface of both PAGP30 and PAGP70 microspheres (Figure 3B). Thus, this result indicated that porous surface of both microspheres was a suitable environment for cell attachment which might improve the recruitment of BMSCs. Additionally, SEM images revealed MS/DBM morphological characteristics that DBM had a three-dimensional microstructure, and that PAGP microspheres remained spherical shape and intact after conjugation (Figure 3C).

### BMSCs proliferation and chondrogenesis on the scaffold

**Figure 4:**
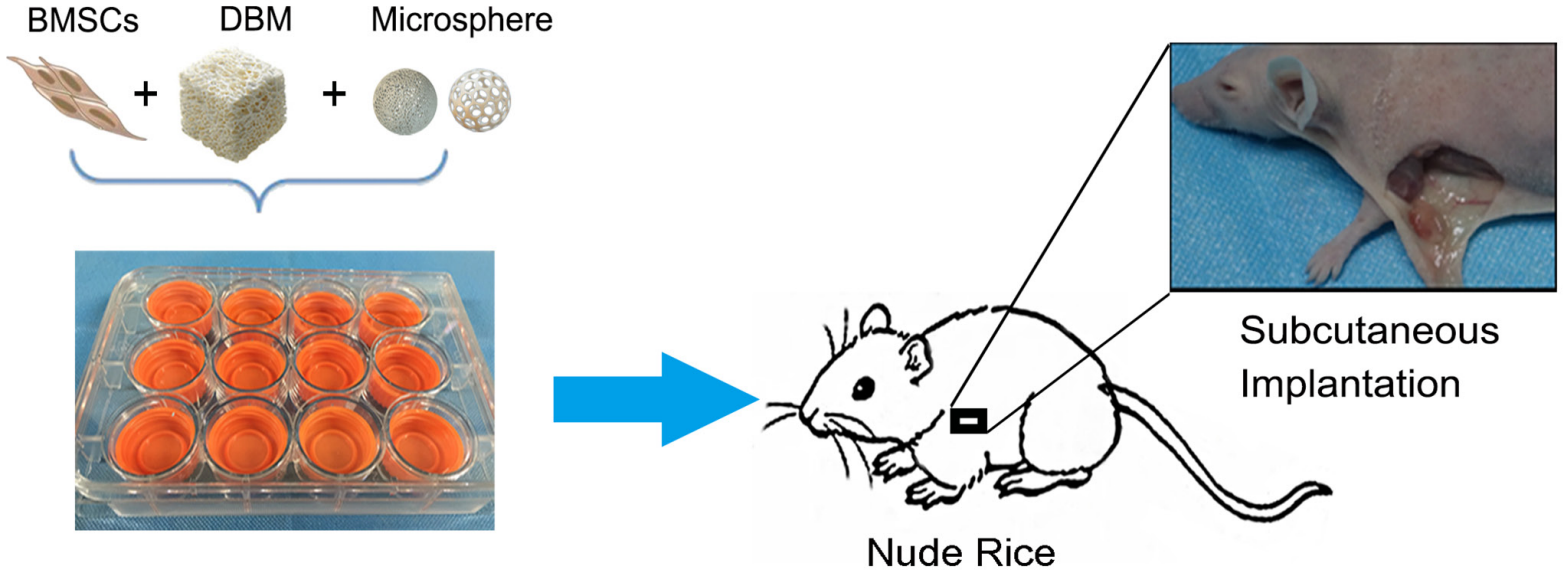
A schematic described animal experimental procedure

**Figure 5:**
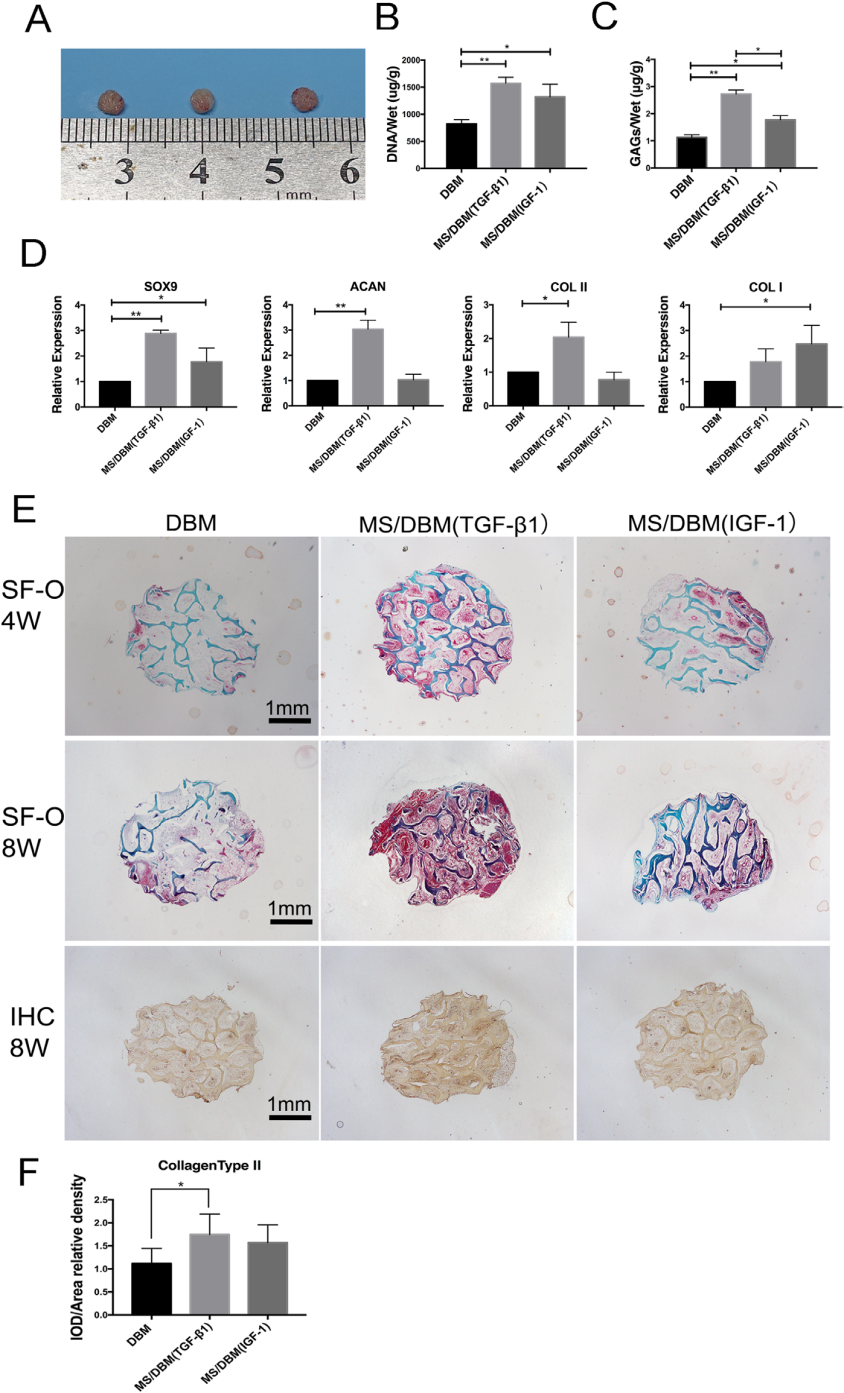
Biological characterization of different scaffolds *in vivo* **(A)** Gross observation when scaffolds were harvested after subcutaneous implantation 8 weeks in nude mice. Left to right: DBM; MS/DBM(TGF-β1) and MS/DBM(IGF-1). **(B)** The DNA content analysis of scaffold after 8 weeks implantation (n=3, ^*^p < 0.05, ^**^p < 0.01). **(C)** The GAGs deposited into scaffolds was analyzed to quantify cartilaginous matrix production by DMMB assay (n=3, ^*^p < 0.05, ^**^p < 0.01). **(D)** Real-time PCR analysis showed genes expression of chondrogenic markers in pure DBM scaffold or MS/DBM scaffolds loaded GFs. Expression was normalized and expressed as fold change compared with the lowest value found in pure DBM group which represented as 1. The Data represent mean ± standard deviation with n = 3. **(E)** SF-O and immunostaining of collagens type II staining of neo-cartilaginous after implanted 4 or 8 weeks (Scale bar=1 mm). **(F)** Values for integrated optical density (IOD) per area of collagens type II from each group (^*^p < 0.05).

The scaffolds were subcutaneously implanted into nude mice for 8 weeks to investigate their biocompatibility and efficacy of chondrogenesis (Figure 4). No signs of inflammatory responses including seroma and infection were observed during sample retrieval. As shown in Figure 5A, translucent scaffolds were transformed into white neo-cartilaginous tissue by gross observation. The DNA content of the construct indicating cell numbers showed that the proliferative capacity of BMSCs on both types of MS/DBM group was higher than DBM alone group (TGF-β1 vs DBM P<0.01, IGF-1 vs DBM P<0.05). Similar to that, the glycosaminoglycan (GAGs) content in MS/DBM (TGF-β1) group also increased significantly (P<0.05) when comparison with DBM group (Figure 5C). These findings indicated that the MS/DBM scaffolds had excellent ability to promote BMSCs proliferation and were more suitable for cartilage matrix production, which might be due to released GFs.

To evaluate the effect of GFs on the chondrogenic phenotype, we conducted quantitative real-time PCR to analyze the expression of genes involved in cartilage matrix synthesis. Compared to DBM alone group (without GFs), the expression levels of Sox9, Acan and Col2a1 were significantly increased in MS/DBM (TGF-β1) groups (Figure 5D). In contrast, MS/DBM (IGF-1) group showed higher expression of Sox9 and type I collagen than control group (P<0.05) which has a synergistic effect on ECM formation at an early stage. These results indicated that implantation using MS/DBM could effectively increase gene expression associated with chondrogensis and TGF-β1 has a positive effect on cartilage-specific matrix formation.

### The histological staining of chondrogenic differentiation for BMSCs cultured on scaffolds *in vivo*

The cartilaginous matrix formation was presented in all types of scaffolds evidenced by positive Safranin-O/Fast green (SF-O) staining after implantation (Figure 5E). The results revealed the structural integrity of DBM (green staining), neo-cartilaginous tissue (red staining) ingrowth and excellent integration with the surrounding tissue. The DBM alone group showed weaker red staining after 4 weeks, while more extensive red staining was presented in both MS/DBM groups, indicating higher level of GAGs content. The process of matrix forming seemed prominent in MS/DBM (TGF-β1) group, and the cells aggregated into a more heterogeneous construction when compared to MS/DBM (IGF-1) group. According to the histological results after 8 weeks, the MS/DBM (TGF-β1) group exhibited most extensive red staining areas from edges to the center. These results were consistent with the above GAGs quantitation results, and it might be due to the fact that TGF-β1 was more suitable for ECM production and PAPG70 microsphere has slower release effect. In addition, immunohistochemistry (IHC) staining showed type II collagen, critical components of cartilage matrix, was significantly increased in all groups at 8 weeks, and the formation of collagen was mostly localized in the pericellular region of MS/DBM (TGF-β1) group (Figure 5D). The relative density of IOD per area value indicated the same trend. These findings indicated that the production of cartilage matrix including collagen and GAGs was significantly increased by GFs released from MS/DBM scaffolds.

### Biomechanical properties of scaffold after subcutaneous implantation in nude mice

**Figure 6:**
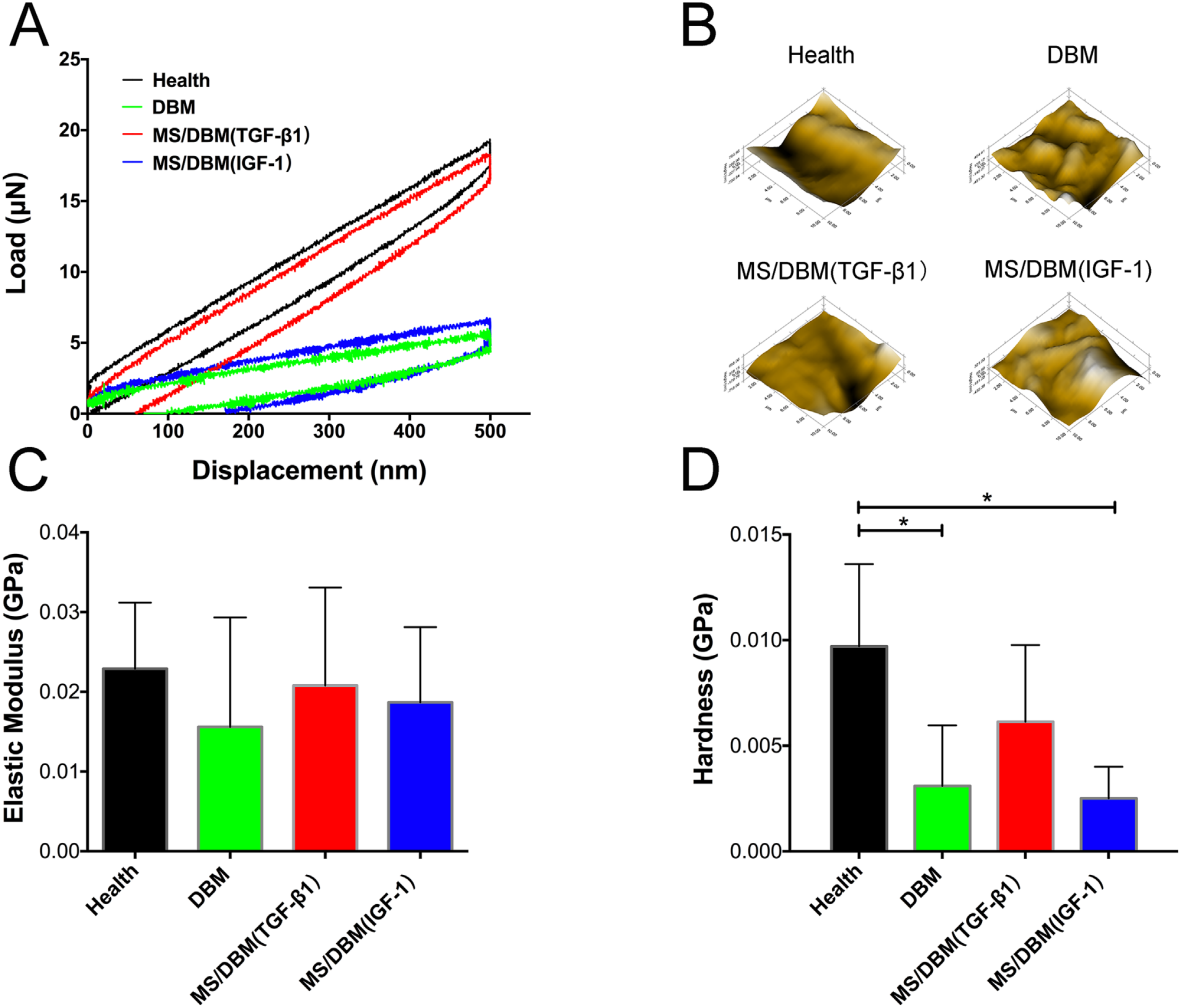
Biomechanical tests of harvested scaffolds **(A)** Representative loaded displacement curves of different groups were recorded within a test range of 500 nm. **(B)** Microscopic geomorphology of the repaired zone was acquired during nanoindentation. **(C, D)** The biomechanical properties of repaired tissue were calculated with the elastic modulus and hardness (^*^p < 0.05).

Finally, we performed nanoindentation assay to evaluate biomechanical properties of the scaffolds in each group. Compared with healthy cartilage, the MS/DBM (TGF-β1) group exhibited remarkable load-displacement curves and hardness. This result further revealed that the biomechanical strength of the scaffold was enhanced by PAGP microsphere with control-released growth factors (Figure 6A, 6D). Moreover, the microscopic geomorphology of indentation zone surface in DBM alone group appeared to be much scraggier and rougher than health cartilage group, while the surface of neo-cartilaginous matrix formation in MS/DBM group was smooth and well integrated (Figure 6B). Taken together, the above biomechanical testing results indicated that the biofunctional MS/DBM scaffold promoted well-organized chondrogenesis, which in turn resulted in a better mechanical strength.

## DISCUSSION

The key to successful regenerative therapies of cartilage repair lies in finding the optimal combination of biomaterials, biofactors and cells [24]. Despite the great advances that have been made in the field of material sciences in mimicking the native tissue environment, most current biomaterials have poor biological effect which restricts their clinical application [25]. In previous reports, we have shown that DBM scaffold could be a desirable biomaterial because its 3D porous structure and biocompatibility [3, 5]. In addition, microsphere-incorporating scaffolds have attracted attention because they can provide excellent initial mechanical properties and the microsphere itself allows controlled release of bioactive molecules [26]. We first time fabricated a composite scaffold with PAGP microspheres and DBM, which combined the effects of two providing multifunctional capability through its characteristics.

In this study, the obtained two novel formulation of PAGP microspheres had different features in chemical composition, hydrophilicity and morphology structures. The occurrence of polymer chain entanglement is a critical factor that determines the morphology of two different microspheres which examined by SEM images (Figure 1). Meanwhile, the polymer weight loss i*n vitro* was also evaluated (Figure 2). It can be efficiently tuned by incorporating hydrophilic or hydrophobic side-substituent groups. Higher glycine ethyl ester content of the polymer caused higher degradation rate due to the ease of water penetration into the matrix (predominantly diffusion due to increased hydrophilicity).

The variation in degradation characteristics of these PAGP microspheres makes it possible to accelerate release based on diffusion and erosion. In our experimental design, a burst release at the initial stage was acceptable because signaling pathways could be activated at the early stage of chondrogenic differentiation. Moreover, the burst release of TGF-β1 encapsulated in PAGP70 microspheres was not very high when compared with the release profile in other publications. Kim et al designed a type of porous chitosan microsphere with cumulative release of almost 80% of total TGF-β1 within 3 days [27]. Lu et al reported TGF-β1 release from PLGA microspheres was about 70% in 5 days [28]. On the other hand, the release of IGF-1 was quite fast within the first few days. The release behavior seems resembling the pattern of IGF-1 in native condition after cartilage damage, that the level of IGF-1 increased during the first 3 days and showed a gradual decrease afterward [22, 23]. Additionally, all synthesized PAGP microspheres have favorable biocompatibility as evaluated by confocal assay. The cells also had a tendency to adhere onto rough surfaces of the microspheres owing to contact guidance phenomenon (Figure 3B). Thus, the surface topological structure can stimulate cell adhesion and might promote cell migration. It also indicated that the PAGP-based microspheres hold an immense potential in the field of control release system.

As introduced above, most of currently available scaffolds contain GFs becomes even more complex because numerous factors have been implicated. Establishment of proper 3D environment for cells condensation and chondrogenesis would garner insight about the underlying mechanisms of cellular interaction, deposition ECM and microsphere-incorporated GFs mode of action which leads to the steering the metabolic activity of cells [14, 29]. Compared to the DBM group by producing hyaline-like cartilaginous matrix, these *in vivo* results demonstrated that the superiority of MS/DBM scaffold to enhance the expression of chondrogenic genes and subsequently leading to neo-cartilaginous matrix deposition (Figure 5). It indicated that the PAPG microspheres released GFs might more closely mimic the native cartilage microenvironment. Hence, this effect could be attributed by released GFs from PAGP microspheres, which constitute a controlled delivery system, it supported and encouraged specific tissue formation at the functional scaffold site.

Fundamentally, native cartilage exhibits good properties in load distribution. Regenerated neo-cartilage should be resistive to tensile and compressive loads *in vivo* [30]. It must be resilient enough to withstand the complex physical forces applied in an articulating joint, while at the same time maintains the stem cell chondronic phenotype, organization of collagen, water uptake by GAGs and provides other matrix components mechanical stiffness [31]. To our knowledge, few reported scaffolds achieved greater initial elastic modulus, compressive hardness and ECM deposition. In the current study, we find that the control-released GFs could improve neo-tissue homeostasis that lead to removal of GAGs entail loss of water and hence increase ultimate biomechanical. In addition, there is still an obvious difference in the aspect of hardness between scaffold groups and the normal cartilage. The composite MS/DBM (TGF-β1) scaffold showed higher hardness, suggesting that GFs reservoir within microspheres influence ECM stiffness and other biomechanical properties, for instance, mediate TGF-β1-driven processes of protein deposition through which this reservoir continuously replenished and depleted. The accumulation, alignment and interaction of ECM proteins further led to enhancing compressive and tensile properties [32]. Because collagen is responsible for the tensile strength of cartilage, low collagen level means that the overall mechanical properties of engineered constructs remain inferior. Therefore, the released GFs are fundamentally during the process of chondrogensis.

The limitation of the present study is that we uncertain the position of implanted microsphere in histology study, which is due to PAGP polymer will dissolve when encountered with alcohol in the process of tissue dyeing. Although green fluorescent protein or other markers might be labelled onto microspheres for tracking after implantation, the labeling time was limited and they might have unfavorable influence on the proliferation and differentiation of BMSCs adhered on the surface of microsphere. Moreover, as the requirements for multiple GF release systems become more prevalent, more sophisticated temporal and spatial control of GFs through PAGP controlled release systems will be focused on further researches.

## MATERIALS AND METHODS

### Synthesis of poly (alanine ethyl ester)_x_(glycine ethyl ester)_y_ phosphazene

Alanine ethyl ester solution and glycine ethyl ester solution was pre-prepared by refluxing the mixture of the amino ethyl ester hydrochloride and triethylamine in THF for 6 h and filtrated for further use, respectively. Linear polydichlorophosphazene (PDCP) was obtained from the bulk polymerization of hexachlorocyclotriphosphazene (HCCP) under vacuum at 250°C for 24 h. After being purified by removing unreacted HCCP, the linear PDCP, containing 0.038 mol of the −PNCl_2_− unit, was dissolved in 200 mL of anhydrous tetrahydrofuran (THF), followed by the addition of alanine ethyl ester (0.076 mol or 0.038 mol) solution in THF (200 ml). The system was allowed to react at 35°C for 24 h under continuous agitation. Subsequently, glycine ethyl ester (0.038 mol or 0.076 mol) solution in THF was added into the system and reacted at 35 °C for another 48 h. Then the reaction was stopped, followed by filtration to obtain a kind of yellowish viscous solution. The solution was concentrated by vacuum rotary evaporation and purified by dialyzed versus THF (four days). The chemical structures of resulted polymers were identified by ^1^H and ^31^P NMR using a Bruker AV600 instrument operated at 400 MHz, in which the ^31^P shifts are relative to an 85% H_3_PO_4_ at 0 ppm as reference. Intrinsic viscosity was measured by a capillary viscosimeter in a water bath thermoset at 30°using THF as solvent. The ^31^P NMR spectrum also shows broad signals at the range of 0 to -2 ppm attributable to the phosphate element on the backbone.

### PAGP microsphere fabrication

The water/oil/water (W_1_/O/W_2_) emulsion method was used for fabricated porous PAGP microspheres [33]. Briefly, 0.6g of PAGP polymer was dissolved in 20ml methylene chloride and Span 80 (1% w/v) was added into the solution (the oil phase). Aqueous solution (2 ml, the W_1_ phase) containing 40 μg/ml rhTGF-β1 (Pepretech, Rocky Hill, USA) or 200 μg/ml rhIGF-I (Pepretech, Rocky Hill, USA) was then added into the oil phase to form the W_1_/O emulsion with the aid of a sonicator (Grant ultrasonic bath XB3, 50−60 Hz, 200 W, UK). The obtained W_1_/O emulsion was transferred into 200 mL of aqueous solution containing poly (vinyl alcohol) (1%, w/v) and Tween 60 (1%, w/v) under continuous agitation (900 rpm). The agitation was continued for at least 4 h to allow the complete evaporation of methylene chloride. Blank PAGP microspheres were prepared in a similar way except no growth factor being added. The microspheres were observed by scanning electron microscope (SEM, S-4800, Japan) and the diameters were measured by Image J analysis software.

### GFs release and microsphere degradation

The GFs-loaded 5 mg PAGP microspheres were immersed in 1 ml PBS and put in a shaker 40 rpm continuously at 37°C. At predetermined time point (1, 2, 4, 7, 10, 14 and 21days), the suspension and the microspheres was separated by centrifugation. After supernatant collected for test, the microspheres were re-suspended with fresh PBS. The concentration of the released TGF-β1 or IGF-I was measured by corresponding ELISAs assay kit (R&D Systems, USA). Percent cumulative release at each time point was normalized to the total encapsulated in microspheres. *In vitro* degradation test for microspheres was conducted in PBS solutions. 20 mg of microsphere was weighed after microspheres had been dried completely in a vacuum oven. Then, biodegradation quantities were determined after they had been dried at the end of time point separate from the solution.

### Cells isolation and culture

Guidelines from Institutional Animal Care and Use Committee at Peking University were followed during all animal procedures. Six-week old Spraguee-Dawley rats were used as source of BMSCs and cells in passage 2 were used for subsequent experiments. The cells were incubated in α-DMEM containing 10% (v/v) FBS and induce differentiation in the chondrogenic differentiation medium (RASMX-90041; Cyagen Biosciences, USA) at 37°C in 5% CO_2_. Flow cytometry was used to assess the specific cell surface antigen markers of BMSCs. Positive markers consisted of CD44 (ab112179) and CD90 (ab225), whereas negative markers consisted of CD34 (ab187284) and CD45 (ab10558) (Abcam Inc, USA).

### Biocompatibility analyses

The density of 6×10^5^ BMSCs and 2 mg PAGP microspheres were seeded onto the bottom of a confocal dish for monolayer co-culture 24 h. After incubation, samples stained with rhodamine phalloidin (160 nM; Cytoskeleton Inc. USA) for 1 h at 37°C, counterstaining using Hoechst 33258 (1:800; Sigma, USA) and observed under confocal microscopy (SP2 inverted microscope; Leica, Germany).

### Preparation of composite MS/DBM scaffolds

The DBM was prepared from porcine femur epiphyses as previously described [5]. Ethylene diamine tetra acetic acid (EDTA; 0.5 M, pH 8.3) was used for demineralization. This DBM scaffold was dissected into a predetermined shape (diameter 4 mm, high 2 mm) and sterilized by cobalt-60. Thereafter, the MS/DBM scaffolds were fabricated by PAGP microspheres solution drop into dry DBM and then vacuum lyophilizing. The total amount of PAGP microsphere per DBM scaffold was 2 mg.

### Scaffold subcutaneous implantation in nude mice

There were 36 female nude mice (Bal/BC, age 6 weeks) randomly divided into DBM, MS/DBM(TGF-β1) and MS/DBM(IGF-1) groups, respectively (n=12, each group). In brief, BMSCs were collected and resuspended at a density of 1×10^7^ cells suspension (100 μ L) which was dropped into each scaffold and incubated at 37 °C for 1 h for cell adhesion. And then, scaffolds cultured in 2 mL of chondrogenic differentiation medium for chondrogenesis 3 days. One subcutaneous pocket was prepared in the armpit of nude mice. Then implanted DBM or MS/DBM scaffold which BMSCs seeded after chondrogenic cultured.

### Neo-cartilage tissue ECM assessment

After 8 weeks, the subcutaneous implanted samples were harvested for gross view, measured weight and digested in a papain solution (Sigma, USA) at 60 °C overnight (n=3 in each group). The content of DNA measured using the Hoechst 33258 fluorometric assay (Polysciences Inc, USA). The fluorescence intensities were then measured at 360 and 460 nm for excitation and emission, respectively. The DNA content was obtained according to a standard curve of calf thymus DNA (Sigma). The DNA contents were normalized to the disk wet weight [34]. The glycosaminoglycan (GAGs) determined using 1,9-Dimethylmethylene blue (DMMB; Sigma St. Louis, MO, USA) dye-binding assay to quantify the sulfated GAGs. The absorbance was measured on a Varioskan Flash instrument at 525 and 460 nm. The GAGs content was determined according to a standard curve based on chondroitin 6-sulfate from shark (Sigma) [35].

### Cartilage-specific gene expression analysis

According to results of microsphere released GFs, some genes related to chondrogensis were upregulated, including SOX-9, ACAN, TGF-β1and IGF-1. Hence, samples for each group were obtained for RNA extraction using TRIzol reagent (Invitrogen, USA). Isolated RNA was reverse-transcribed and real-time PCR analysis was performed using ABI 7300 step one plus RT-PCR System (Applied Biosystems, USA) with SYBR Green PCR Master Mix (Toyobo, Osaka, Japan). The sequences of the PCR primers are presented in (Table 2). Moreover, the expression level of 18sRNA was used as internal control. The relative expression changes in these target genes were quantified by 2-ΔΔCt method.

**Table 2:** Real-time PCR primers

Gene	Forward primer (5’–3’)	Reverse primer (5’–3’)
18s RNA	GTAACCCGTTGAACCCCATT	CCATCCAATCGGTAGTAGCG
SOX9	CAAGAAAGACCACCCGGACT	GCCTTGAAGATGGCGTTGGG
ACAN	CCTACCAGGACAAGGTCTCG	ACCTCACAGCGGTAGATCCC
COL II	CACCGCTAACGTCCAGATGAC	GGAAGGCGTGAGGTCTTCTGT
COL I	TGGTGGATGCTCTCAGTTCGTGT	TGGTGGATGCTCTCAGTTCGTGT

### Histological evaluations

At 4 and 8 weeks, the specimens were fixed in 4% paraformaldehyde (pH = 7.4) for 48 h at room temperature, dehydrated in a graded ethanol series and embedded in paraffin. Sections were sagittal cut 6 μm-thick and stained with SF-O or IHC with type II collagen antibodies (Calbiochem Cat No.: CP18-100UG; USA) according to standard protocols. Moreover, IHC analyses were utilized to conduct semi-quantitative study of the differences of collagen contents within the implants. Briefly, 10 digital images were captured by an Olympus BX-51 microscope. Integrated optical density (IOD) value and area of each image were measured with Image-Pro Plus 6.0 software (Media Cybernetics). The descriptors of relative density (IOD per area) was measured to semi-quantify the deposition of collagen II.

### Nanoindentation assessment biomechanical

Biomechanical analysis of implanted scaffolds using nanoindentation after 8 weeks. Samples (n=3) were isolated from the central part of scaffold. Hydration was maintained utilizing a circumfluent PBS solution at room temperature. All indentations were performed using the TriboIndenter (Hysitron Inc. USA) with a 400-mm radius curvature conospherical diamond probe tip. A trapezoidal load function was applied to each indent site with loading (10 s), hold (2 s), and unloading (10 s). Indentations were force-controlled to a maximum indentation depth of 500 nm. Meanwhile, the microscopic geomorphology of the indentation zones was captured using micro-scanning apparatus.

### Statistical analysis

All the data were expressed as mean ± standard deviation and represented at least three independent experiments. Statistical analyses using one-way analysis of variance by SPSS 20 software. P < 0.05 was considered statistically significant.

## CONCLUSIONS

In this study, two kinds of PAGP microspheres with different physicochemical properties were prepared by changing the molecular ratio of side-substituents group. They possessed different surface roughness and hydrolytic degradation profiles. The MS/DBM scaffold led to significantly more cartilaginous tissue after subcutaneous implantation. Meanwhile, this biocompatible scaffold could potentially improve biomechanical properties. It is reasonable for us to believe that PAGP MS/DBM scaffolds could be a valuable biomaterial in regenerative engineering due to its structure and function properties.
